# Spontaneous fermentation of Tunisian dromedary milk: VASE™-GC–MS volatile profiling, physicochemical and mineral composition, bioactivities, and lactic acid bacteria enumeration

**DOI:** 10.3389/fnut.2026.1897549

**Published:** 2026-09-09

**Authors:** Ahmed Neji, Amna Chahbani, Zeineb Jrad, Olfa Oussaeif, Samia Kdidi, Mouhamed Hammadi, Fouad Achemchem, Khaoula Belguith, Doriane Martin, Halima El-Hatmi

**Affiliations:** 1 Livestock and Wildlife Laboratory LR16IRA04, Arid Land Institute of Medenine, University of Gabes, Medenine, Tunisia; 2 Food Department, Higher Institute of Applied Biology of Medenine, University of Gabes, Medenine, Tunisia; 3 Bioprocess and Environment Team, LASIME Lab., Agadir Superior School of Technology, Ibn Zohr University, Agadir, Morocco; 4 Analytical, Valorization and Food Safety Laboratory, National Engineering School of Sfax, Sfax, Tunisia; 5 Physiopathology, Food and Biomolecules Laboratory (LR17ES03), Higher Institute of Biotechnology Sidi Thabet, University of Manouba, Sfax, Tunisia; 6 Laboratoire de Recherche en Génie Industriel Alimentaire (LRGIA), Université de Lorraine, Nancy, France; 7 Research Unit BIODYMIA, Université Lyon 1, ISARA, Bourg-en-Bresse, France

**Keywords:** antimicrobial activity, antioxidant activity, dromedary camel milk, spontaneous fermentation, kinetics, organic acids, HS- VASETM - GC-MS

## Abstract

**Introduction:**

Fermented dromedary camel milk is a traditional food in arid and semi-arid regions, yet the kinetics of spontaneous fermentation remain insufficiently characterized, particularly for Tunisian milk. This study investigated the 72 h spontaneous fermentation of raw Tunisian dromedary milk using an integrated kinetic approach combining physicochemical, compositional, microbiological, and bioactivity analyses.

**Methods:**

Raw milk was incubated at 37 °C without starter culture and sampled at 0, 12, 24, 48, and 72 h in three independent biological replicates. Physicochemical parameters, mineral composition, sugars, organic acids, volatile compounds by VASE™-GC-MS, fatty acids, lactic acid bacteria (LAB) counts, antioxidant activity, and antimicrobial activity were evaluated.

**Results:**

Fermentation induced progressive acidification, as reflected by a decrease in pH and an increase in titratable acidity, together with increased viscosity and reduced water activity. Total solids and protein content decreased slightly, whereas fat and ash contents remained relatively stable. Sodium and magnesium concentrations increased during fermentation, while calcium and potassium decreased and iron remained stable. Lactose consumption was accompanied by the accumulation of lactic and succinic acids, which is consistent with active microbial metabolism. VASE™-GC-MS analysis revealed time-dependent changes in the volatile profile throughout fermentation. The lipid fraction remained dominated by long-chain fatty acids, mainly C16C18. LAB counts increased from 4.30 to 8.77 log CFU mL^−1^, while antioxidant and antimicrobial activities were significantly enhanced over time.

**Conclusion:**

Overall, spontaneous fermentation improved the technofunctional, antioxidant and antimicrobial properties, highlighting its potential as a traditional functional food.

## 1 Introduction

Global dairy systems are increasingly challenged by climate change, water scarcity, and land degradation, prompting renewed interest in livestock species that are naturally adapted to arid and semi-arid environments (1–3). In this context, the dromedary camel represents a strategic dairy resource in dryland regions, including southern Tunisia, where it contributes to food security, livelihood resilience, and the valorization of marginal ecosystems (2, 4). Beyond fresh consumption, camel milk is also traditionally processed into fermented products; particularly in areas where refrigeration and organized milk collection remain limited (4–6). Under such conditions, spontaneous fermentation is not only a practical means of preservation, but also an integral part of local food systems and traditional processing practices.

Camel milk exhibits distinctive compositional and functional features that differentiate it from bovine milk, including differences in protein organization, whey protein profile, lipid fraction, mineral balance, and bioactive constituents (2, 3, 7–10). These characteristics contribute to its nutritional value and have been associated with several health-promoting properties, including antioxidant and antimicrobial potential, particularly through milk proteins and derived peptides (3, 8–11). At the same time, they strongly influence the technological behavior of camel milk, including its coagulation, gelation, heat stability, and fermentation performance (2, 4, 12, 13). Consequently, fermentation patterns established for bovine milk cannot be directly extrapolated to camel milk, whose specific physicochemical matrix presents both constraints and opportunities for processing.

In traditional settings, camel milk fermentation generally occurs without the addition of standardized commercial starter cultures and instead relies on the activity of indigenous microbiota originating from raw milk, utensils, and the surrounding environment (5, 14–17). This spontaneous process drives a cascade of biochemical and microbiological transformations that shape product acidity, texture, aroma, and functional properties over time (5, 17–19). Although previous studies have documented the presence and diversity of autochthonous lactic acid bacteria (LAB) in camel milk and fermented camel milk products (14–17, 20–22), much less is known about the kinetics of these transformations during spontaneous fermentation, particularly in Tunisian dromedary milk.

Despite the growing body of literature on camel milk and its fermented products, several specific aspects of spontaneous fermentation remain insufficiently characterized. First, the time-course evolution of physicochemical parameters, mineral composition, sugar utilization, organic acid accumulation, volatile compound formation, fatty acid profile, LAB growth, and bioactivity changes has rarely been documented within a single integrated experimental design, particularly for Tunisian dromedary milk. Second, the dynamics of mineral redistribution during fermentation, especially for trace elements such as iron, have received limited attention, despite their nutritional relevance in arid regions where camel milk is consumed. Third, although the volatile profile of fermented camel milk has been partially described in final products, the kinetic evolution of volatile compounds during spontaneous fermentation has been seldom investigated, and few studies have applied integrated GC–MS-based approaches under conditions reflecting traditional fermentation practices.

Volatile organic compounds (VOCs) play a central role in the sensory quality, fermentation monitoring, and authentication of fermented dairy products (23–25). In fermented milks, the formation of VOCs reflects the interplay between microbial metabolism, substrate availability, and matrix conditions, making VOC profiling a powerful tool for tracking fermentation dynamics. However, despite their relevance, integrated GC–MS-based volatilomic studies of spontaneously fermented camel milk remain scarce, and existing reports have largely focused on the volatile composition of final products rather than on the kinetic evolution of volatile compounds during fermentation (24, 25). To address this gap, the present study applied a VASE™-GC–MS approach, which combines vacuum-assisted sorbent extraction with gas chromatography–mass spectrometry to enhance extraction efficiency, sensitivity, and detection of low-abundance volatile compounds (26–29). This approach is particularly suitable for monitoring the time-dependent dynamics of volatile compounds during spontaneous fermentation of dairy matrices.

Addressing this gap is important for both scientific and practical reasons. A better understanding of spontaneous fermentation kinetics could help identify key fermentation stages associated with improved functional quality while also providing a basis for the valorization and more consistent processing of traditional camel milk products. Therefore, the aim of the present study was to investigate the 72 h spontaneous fermentation of Tunisian dromedary milk using an integrated kinetic approach. Changes in physicochemical characteristics, mineral composition including iron, sugars, organic acids, volatile compounds determined by VASE™-GC–MS, fatty acids, LAB counts, antioxidant activity, and antimicrobial activity were monitored throughout fermentation. We hypothesized that spontaneous fermentation would induce marked time-dependent transformations across these parameters and that their combined analysis would help identify fermentation stages associated with improved technofunctional and bioactive properties. To the best of our knowledge, no previous study has integrated, within a single 72 h spontaneous fermentation kinetic framework, the simultaneous monitoring of physicochemical parameters, mineral composition (including iron), sugar utilization, organic acid accumulation, VASE™-GC–MS volatile profiling, fatty acid composition, LAB growth, and antioxidant and antimicrobial activities of Tunisian dromedary camel milk. By integrating these complementary dimensions, this study provides new insight into the interconnected biochemical and microbiological processes underlying traditional camel milk fermentation and supports the scientific valorization of this product as a functional fermented food.

## 2 Materials and methods

### 2.1 Milk samples preparation and pre-fermentation handling

Raw camel milk samples were obtained from dromedary camels (*Camelus dromedarius*) and reared at the experimental farm of the Livestock and Wildlife Laboratory (LR16IRA04), Arid Land Institute of Medenine, southern Tunisia (latitude ~33.3°N, longitude ~10.5°E). The animals were healthy, lactating she-camels managed under the standard extensive pastoral system of the experimental station, with free access to natural pasture and water and supplemental feeding according to physiological needs.

The collection was performed manually under hygienic conditions during the morning milking session, from four lactating dromedary camels at mid-lactation stage, after discarding the first streams of milk to reduce microbial contamination. For each milk collection, individual milk samples were immediately combined into a single pooled batch to obtain a representative composite sample and to minimize inter-animal variability.

The pooled raw milk was transferred into sterile glass containers and transported to the laboratory under refrigerated conditions (4 ± 1 °C) within 5 min after milking, and processed immediately to preserve the indigenous microbiota and to avoid uncontrolled microbial growth.

The fermentation experiment was performed using three independent biological replicates, each corresponding to a separate milk collection and fermentation batch carried out on different days.

Spontaneous fermentation was initiated immediately upon arrival at the laboratory. The pooled raw milk was distributed into sterile glass containers and incubated at 37 °C for 72 h, without the addition of any starter culture, to allow the natural development of indigenous microbiota originating from raw camel milk, as commonly practiced in traditional spontaneous fermentation of dromedary milk in North African production systems (6, 14, 19, 30).

Samples were collected aseptically at five fermentation time points: 0 h (T0, raw milk before incubation), 12 h (T12), 24 h (T24), 48 h (T48), and 72 h (T72). At each time point, aliquots were taken for physicochemical, mineral, sugar, organic acid, volatile compound, fatty acid, LAB, antioxidant, and antimicrobial analyses.

Experimental design with biological replicates. The entire fermentation experiment was performed using three independent biological replicates, each corresponding to a separate milk collection and fermentation batch carried out on different days under identical conditions. This experimental design was specifically chosen to capture biological variability associated with separate animal milking sessions and to ensure the robustness and reproducibility of the kinetic data.

### 2.2 Physicochemical analysis

The pH value was measured at 20 °C using a Jenway pH meter (Model 3,510, Staffordshire, United Kingdom) previously calibrated, while the acidity was determined by titrating 10 mL of milk or whey with a solution of sodium hydroxide NaOH (0.1 N) in the presence of phenolphthalein (0.5%). The acidity is expressed in Dornic degrees (°D), following standard dairy analytical methods. Total solid, ash, protein, and total fat contents were measured using gravimetric drying, incineration, Kjeldahl nitrogen, and solvent extraction methods, respectively, following established procedures in milk analysis. All analytical procedures followed internationally recognized standards, including AOAC Official Methods of Analysis and ISO 1211:2010 for fat determination and ISO 8070:2007 for mineral analysis. Viscosity was measured using a rotational viscometer (Brookfield, model DV-E, MA, USA), and water activity (a_w) was determined using a water activity meter (LabSwift-aw, Novasina, Switzerland). All analytical determinations were performed in analytical triplicates for each biological replicate.

### 2.3 Determination of organic acids and sugars

Organic acid analysis was performed after sample defatting by centrifugation followed by filtration of the deproteinized supernatant through a 0.45 μm filter. Organic acids (oxalate, citrate, quinic acid, lactate, formate, acetate, succinate) were quantified by high-performance liquid chromatography (HPLC) using a Shimadzu ultra-fast LC system (LC-20 AD XR binary pump, SIL-20 AC XR autosampler, CTO-20 AC column oven, DGU-20AS degasser, SPD-M20A diode array detector; Shimadzu, Kyoto, Japan). Separation was achieved on an Agilent Hi-Plex H column (7.7 × 300 mm, 8 μm particle size) maintained at 50 °C, with isocratic elution using 0.1 M H₂SO₄ aqueous mobile phase at 0.6 mL min^−1^. Samples (10 μL injection volume, filtered through 0.45 μm membrane) were monitored at 210 nm wavelength using Shimadzu LabSolutions v5.42 software. Calibration curves were constructed from individual standards (Sigma-Aldrich, >98% purity) at 10–1000 μg mL^−1^ (r^2^ ≥ 0.999, 6 points), with limits of detection (LOD) 0.1–0.5 mg L^−1^ and limits of quantification (LOQ) 0.3–1.5 mg L^−1^. Method validation confirmed 95–102% recovery in fermented milk matrix, intra-day repeatability <3% RSD, and inter-day <5% RSD (*n* = 6). Results were expressed as mg L^−1^. Glucose and lactose were analyzed in parallel using refractive index detection (RI), with concentrations expressed as g L^−1^. Temporal changes in carbohydrate consumption and organic acid production were monitored from T0 to T72 during spontaneous fermentation.

### 2.4 Determination of volatile organic compounds (VASE™-GC–MS)

#### 2.4.1 Extraction procedure of VOCs

VOC extraction was performed in 20 mL glass vials using a vacuum-assisted sorbent extraction system (VASE™). Volatile compounds were collected using headspace sorbent pens™ packed with Tenax^®^ TA (35/60 mesh; model SP-HSP-T3560, Entech Instruments, Simi Valley, CA, USA, whose local supplier is Revolab, Cergy, France), a porous polymer widely recognized for its high adsorption capacity toward a broad range of VOCs. Subsequently, 1 g of frozen sample was introduced into the vials, a sorbent pen™ was inserted, and the vial was sealed using dedicated caps designed to accommodate the sorbent pen™ while maintaining airtight conditions. After assembly, air evacuation was conducted using a vacuum membrane pump for approximately 15 s (the time required for the vacuum to reach 28 in Hg in the vial) to maximize the extraction. Control experiments without vacuum assistance were also performed. The extraction of volatile compounds was performed for 1 h at room temperature (18 °C). The samples were used only once for extraction.

#### 2.4.2 VOC analysis by GC–MS

VOC analysis was carried out using a TRACE™ 1,310 gas chromatograph coupled with an ISQ 7000 single quadrupole mass spectrometer (Thermo Fisher Scientific Inc., Waltham, MA, USA). Separation was performed on a Rtx-502.2 column (60 m, 0.32 mm i.d., 1.8 μm film thickness; Restek, Bellefonte, PA, USA). VOCs were desorbed from the sorbent pen™ under the following operating conditions: desorber temperature 70 °C, preheat 0.20 min at 150 °C, desorption for 10 min at 260 °C, bake out for 5 min at 280 °C, back flush for 10.5 min at 100 °C followed by cooling. Injection was performed in split mode (1:10). The GC oven temperature program was set as follows: initial temperature of 40 °C for 2 min; then ramped at 5 °C min^−1^ up to 130 °C, and then at 30 °C min^−1^ up to 235 °C (maintained for 2 min). Helium was used as carrier gas at a constant flow rate of 1 mL min^−1^. The GC/MS transfer line was set at 250 °C, and the mass spectrometer was operated in full-scan mode over an m/z range of 40–450 atomic mass units (a.m.u.) at a scan rate of 0.2 scans s^−1^. All experiments were conducted in triplicate.

#### 2.4.3 VOC data processing and identification

Raw GC–MS data files were processed using the Thermo Scientific™ Chromeleon™ Chromatography Data System (CDS) software (Thermo Fisher Scientific, Waltham, MA, USA). Chromatographic peak integration was performed manually to ensure consistent baseline assignment and accurate peak area determination, particularly for trace-level compounds and partially co-eluting peaks.

The identification of volatile organic compounds (VOCs) was validated according to a dual identification criterion. First, the experimental mass spectra were compared with reference spectra from the NIST (National Institute of Standards and Technology) Mass Spectral Library. Second, identification was confirmed by comparing the experimental retention indices (RI) with those of authentic pure standards injected under identical chromatographic conditions. Only compounds matching both identification criteria were retained for further analysis.

**Figure 1:**
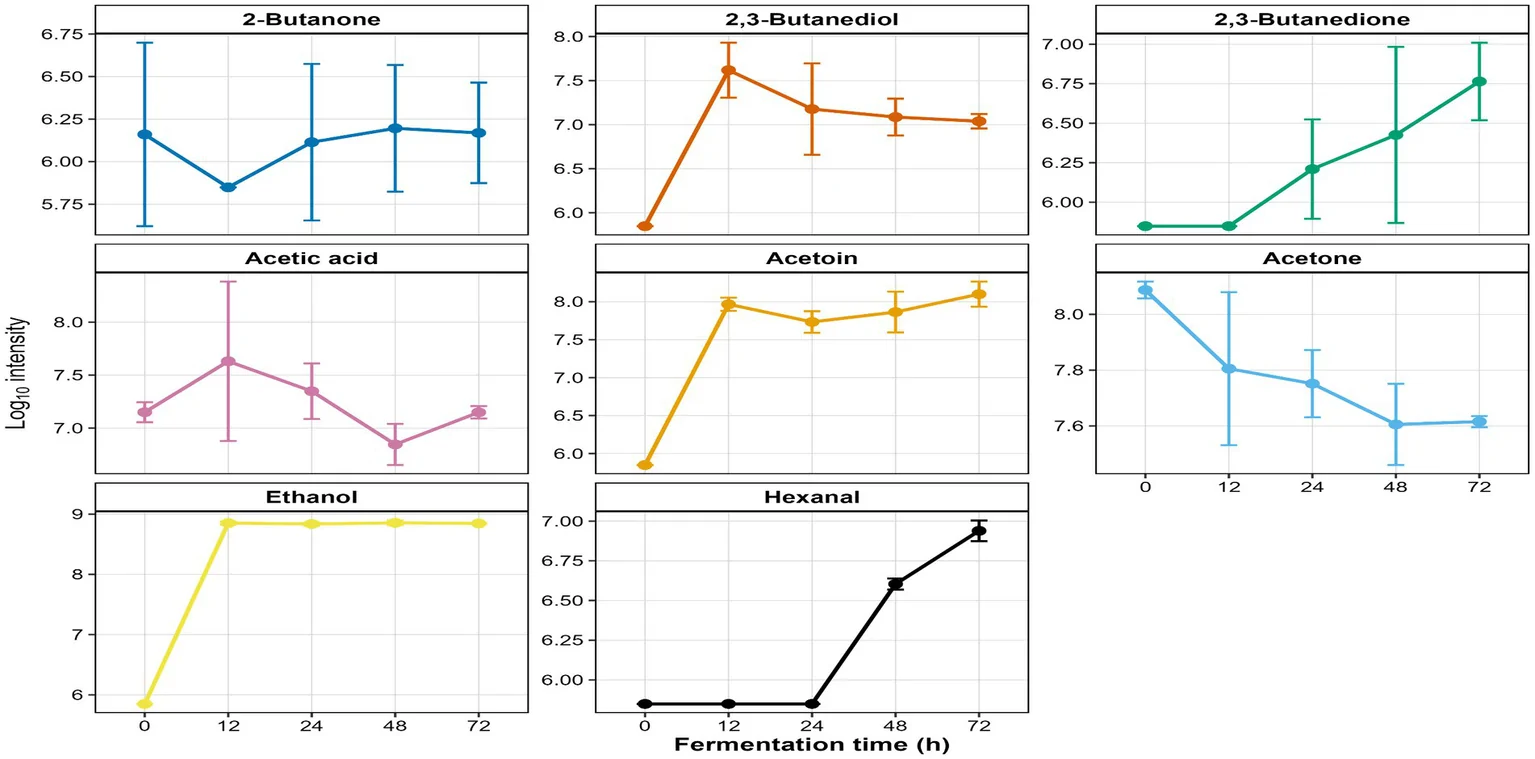
Volatile compounds profile of dromedary camel milk during 72 h of fermentation (VASE-GC–MS; mean SD, *n* = 3): compound 1: Ethanol–compound 2: Acetone–compound 3: Acetic acid–compound 4: 2,3-Butanedione–compound 5: 2-Butanone–compound 6: Acetoin–compound 7: 2,3-Butanediol–compound 8: Hexanal.

For each identified compound, peak areas were extracted, and log₁₀-transformed peak areas were used for the graphical representation of VOC profiles (Figure 1) to reduce the impact of high-intensity peaks and to facilitate visual comparison of compounds with different abundance ranges, as commonly performed in volatilomic studies of fermented dairy products.

For each fermentation time point (T0, T12, T24, T48, T72), data from three independent biological replicates were averaged and expressed as mean ± standard deviation (SD). Statistical analysis of VOC data was performed on log₁₀-transformed peak areas following the same statistical framework described in Section 2.9 (one-way ANOVA + Tukey’s HSD test, *p* < 0.05).

### 2.5 Determination of fatty acid composition

Milk fat was extracted from both fermented and non-fermented dromedary camel milk samples using the Folch extraction method, which involves a chloroform: methanol mix in a 2:1 ratio. To convert the extracted lipids into fatty acid methyl esters (FAME), acid-catalyzed transesterification was performed with BF₃-methanol (14%, 2 mL from Sigma-Aldrich) at 80 °C for 1 h, adding nonadecanoic acid (C19:0, 1 mg mL^−1^) as an internal standard. The separation and quantification of FAME were carried out using a GC-FID system from Agilent, equipped with an Rtx-2330 capillary column (100 m × 0.25 mm i.d., 0.20 μm film thickness). The injector temperature was set to 260 °C in split mode with a split ratio of 1:100 and a total flow of 35 mL min^−1^, using helium as the carrier gas at a flow rate of 1.0 mL min^−1^ and a linear velocity of 20 cm s^−1^. The oven temperature program started at 140 °C (held for 0 min), then ramped to 210 °C at a rate of 20 °C min^−1^, and held for 11 min. The FID detector operated at 260 °C. Individual fatty acids ranging from C4:0 to C20:0, including both saturated (e.g., C18:0 and C20:0) and unsaturated (e.g., C18:1 and C20:1) types, were identified by comparing their retention times to those of a FAME 37 mix standard from Supelco, with a correlation coefficient (r^2^) greater than 0.999. For quantification, the internal standard method with C19:0 was used, and quality control was ensured through blanks, duplicates (with a relative standard deviation of less than 5%), and control milk samples. The results are reported as a percentage of the total identified FAME.

### 2.6 Determination of antioxidant activities

Three complementary assays were used to evaluate antioxidant capacity: metal-chelating activity, ABTS radical-scavenging activity, and *β*-carotene bleaching inhibition. For all three antioxidant assays, fermented camel milk samples were tested at seven concentrations (0.1, 0.2, 0.4, 0.6, 0.8, 1.0, and 1.5 mg mL^−1^). The IC₅₀ values, defined, as the sample concentration required inhibiting 50% of the activity, were calculated from dose–response curves by nonlinear regression analysis.

#### 2.6.1 Ferrous ion chelating assay (FCA)

The FCA was conducted according to the protocol established by Chan et al. (2007) (31). A 2 mM solution of freshly prepared ferrous sulfate hydrate (FeSO₄·xH₂O) and a 5 mM ferrozine solution were both diluted to 1/20 of their original concentration prior to use. In the procedure, 1 mL of the diluted FeSO₄·xH₂O was combined with 1 mL of the water-soluble extracts. Following this, 1 mL of the diluted ferrozine was added to the mixture, which was then incubated for 10 min at room temperature. The absorbance was measured at a wavelength of 562 nm using a spectrophotometer, with distilled water serving as the blank. The control absorbance was determined in a similar manner, substituting the supernatant with distilled water. The ferrous ion chelating activity (FCA) was calculated as:

FCA(%)=[(A_control−A_sample)/A_control]×100

Where A_control and A_sample are the respective absorbance of the control and sample at 562 nm.

#### 2.6.2 ABTS radical scavenging activity

ABTS (2,2′-azinobis[3-ethylbenzothiazoline-6-sulfonic acid]) radical scavenging activity was determined using the method described by Re et al. (1999) (32), with minor modifications. An ABTS radical cation stock solution was prepared by dissolving 7 mM ABTS in 2.45 mM potassium persulfate buffer with an equal volume (1:1). This mixture was kept in the dark for 12–16 h at room temperature. The ABTS radical cation stock solution was subsequently diluted with sodium phosphate buffer (5 mM, pH 7.4) to obtain a working solution with a final absorbance of 0.70 ± 0.05 at 734 nm. A 0.25 mL volume of each sample at various concentrations was added to 1 mL of the diluted ABTS working solution, and the mixture was incubated in the dark for 10 min at room temperature. Absorbance was measured at 734 nm using a UV–Visible spectrophotometer (Cecil CE 2041, Cambridge, UK). A control was prepared in the same manner as the sample, except that distilled water was used instead of the sample. Ascorbic acid was used as a positive control. The percentage of ABTS radical scavenging activity was calculated as:

$$\begin{array}{l} \text{ABTS radical}\\ \;\text{inhibition}\;(\%)=[\begin{array}{l} \left(A\_\text{control}-A\_\text{sample}\right)\\ /A\_\text{control} \end{array}]\times 100 \end{array}$$

Where A_control and A_sample are the respective absorbance of the control and sample at 734 nm.

#### 2.6.3 Β-Carotene bleaching test (BCBT)

A modified version of the method established by Koleva et al. (33) was utilized to perform the β-carotene bleaching test. Initially, 0.5 mg of β-carotene was dissolved in 1 mL of chloroform. To this solution, 25 μL of linoleic acid and 200 μg of Tween 40 were added. The chloroform was then evaporated under vacuum at a temperature of 40 °C. Following this, 50 mL of oxygenated ultra-pure water was added to the residue, and the mixture was vigorously shaken to form an emulsion. This emulsion was freshly prepared prior to each experiment. Then, 150 μL of sample were added to 150 μL of the *β*-carotene-linoleic acid emulsion. The plate was incubated at 50 °C for 60 min. The absorbance was measured at 470 nm using a microplate spectrophotometer (Thermo Scientific Multiscan Go). The control was performed in the same way, except that distilled water was used instead of the sample. The percentage of antioxidant activity was calculated as follows:

$$\text{Antioxidant activity}\;(\%)=[1-(\begin{array}{l} \left(A_{o}-\mathrm{At}\right)\\ /(A_{o}^{\prime }-A^{\prime }t) \end{array})]\times 100$$

Where A₀ and At are the respective absorbance of the sample recorded before and after incubation, and A’₀ and A’t are the absorbance of the control recorded before and after incubation, respectively.

### 2.7 Enumeration of lactic acid bacteria (LAB)

LAB were enumerated on selective Man-Rogosa-Sharpe MRS agar (BIOKAR REF BK089HA) and M17 agar (BIOKAR REF088HA) after incubation at 37 °C for 48 h, with results expressed as colony-forming units per milliliter (CFU mL^−1^). Representative colonies were confirmed as catalase-negative and Gram-positive LAB through standard biochemical tests, ensuring selective enumeration of the target microbial group. Sampling was carried out at T0, T12, T24, T48, and T72, following procedures commonly used in fermented dromedary camel milk studies (14, 15, 22).

### 2.8 Determination of antimicrobial activity

Antimicrobial activity was tested using the agar well diffusion assay on four indicator bacteria: *Escherichia coli* ATCC 25922, *Staphylococcus aureus* ATCC 25923, *Bacillus cereus* ATCC 11778, and *Salmonella Typhi* ATCC 19430, which are known to cause foodborne illnesses (34). Bacterial suspensions were adjusted to a 0.5 McFarland standard turbidity and spread onto Mueller-Hinton agar plates. Wells of 6 mm diameter were punched into the agar and filled with 50 μL of cell-free supernatants of fermented dromedary camel milk obtained after centrifugation and filtration, according to the method described by Benkerroum et al. (2003) (14). Plates were incubated at the optimal temperature for each test strain for 18–24 h, and inhibition zones were measured in millimeters using a calibrated digital caliper.

In the present study, antimicrobial activity was assessed after pH neutralization to exclude the effect of acidity as the sole inhibitory factor. Fermented milk samples were centrifuged at 8,000 × g for 15 min at 4 °C. The pH of the resulting supernatants was adjusted to 7.0 ± 0.1 using 1 M NaOH, and the neutralized supernatants were subsequently filter-sterilized through 0.22 μm membranes prior to use in the agar well diffusion assay.

In parallel, wells containing sterile distilled water were included as a negative control to verify that any observed inhibition was attributable to the fermented camel milk supernatants rather than to the diffusion procedure itself.

### 2.9 Statistical analysis

Experimental design and replicates. The spontaneous fermentation experiment was carried out using three independent biological replicates, each corresponding to a separate batch of raw camel milk fermented under identical conditions (37 °C, 72 h). For each biological replicate and each fermentation time point (T0, T12, T24, T48, T72), samples were collected aseptically and analyzed. All analytical determinations (physicochemical parameters, mineral composition, sugars, organic acids, volatile compounds, fatty acids, LAB counts, antioxidant activities, and antimicrobial activities) were performed in analytical (technical) triplicates for each biological replicate. The mean value of the technical triplicates was used as the representative value for each biological replicate, and the final results were expressed as mean ± standard deviation (SD) of the three biological replicates (*n* = 3).

Parametric statistical analysis. For each parameter, differences across fermentation time points were assessed by one-way analysis of variance (ANOVA). When significant differences were detected, post-hoc multiple comparisons were performed using Tukey’s honestly significant difference (HSD) test. Different lowercase superscript letters in tables and figures indicate significant differences between fermentation time points (*p* < 0.05).

Statistical treatment of VOC data. For VOC analyses, statistical comparisons were performed on log₁₀-transformed peak areas to stabilize variance and to normalize the distribution of compounds with widely varying abundances, as commonly applied in volatilomic studies of fermented dairy products. The log₁₀-transformed data were then analyzed using the same ANOVA + Tukey HSD framework described above. Compounds detected in fewer than two of the three biological replicates at a given time point were considered as below the reliable detection threshold and were excluded from statistical analysis.

Software and significance threshold. All statistical analyses were performed using IBM SPSS Statistics (version 26, IBM Corp., Armonk, NY, USA). Statistical significance was set at *p* < 0.05.

## 3 Results

### 3.1 Physicochemical parameters

**Figure 2:**
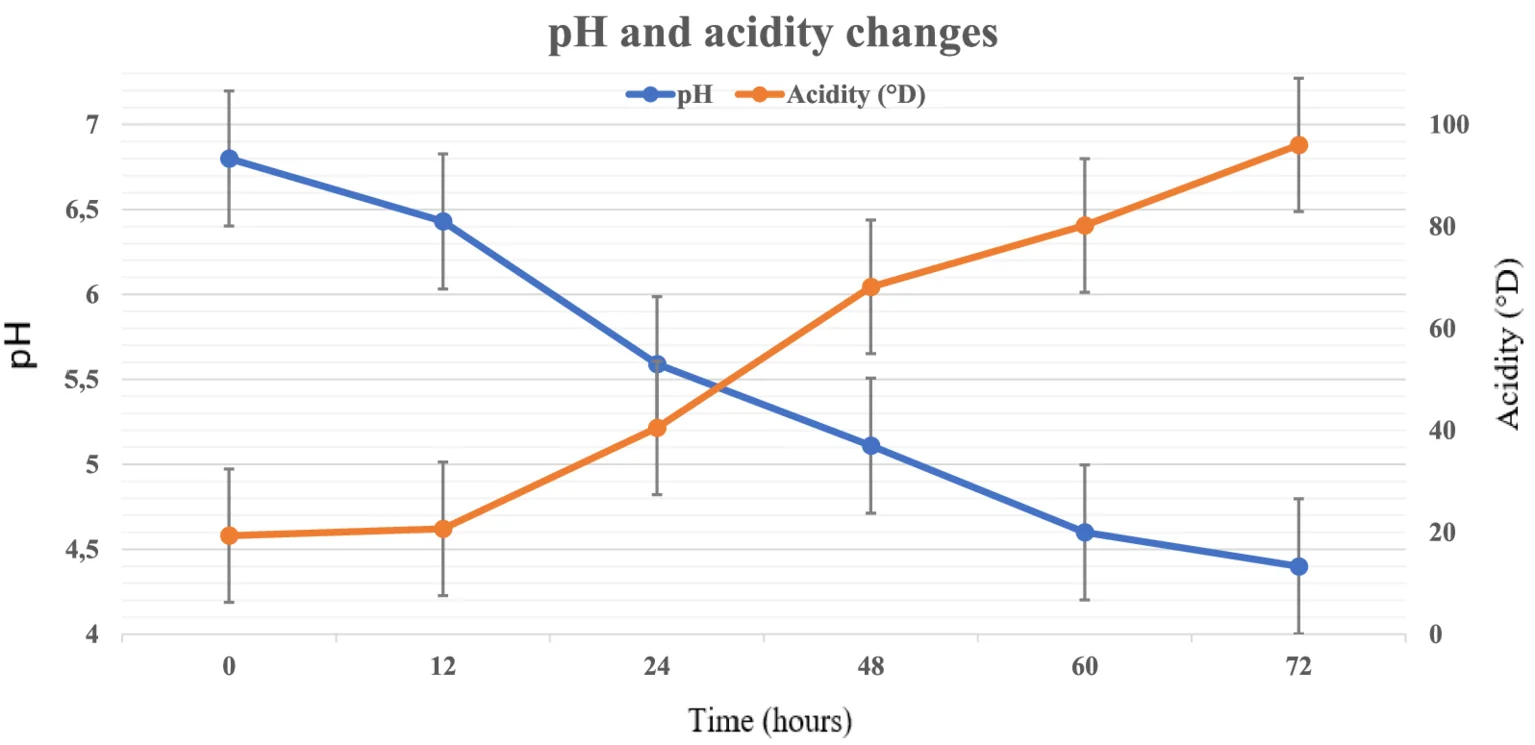
pH and acidity changes over time of dromedary camel milk during spontaneous fermentation.

The physicochemical parameters of Tunisian dromedary camel milk evolved significantly during the 72 h spontaneous fermentation (Table 1; Figure 2).

**Table 1:** Variation in the physicochemical constituents of dromedary camel milk during 72 h of fermentation.

Parameters		Fermentation time in hours				
		T0	T12	T24	T48	T72
Dry matter (g L^−1^)		122.86 ± 0.17^a^	121.90 ± 0.25^ab^	121.30 ± 0.30^b^	120.90 ± 0.35^bc^	120.67 ± 0.37^c^
Ash (g L^−1^)		6.38 ± 0.18^a^	6.37 ± 1.03^a^	6.21 ± 0.47^ab^	6.13 ± 1.11^ab^	6.01 ± 0.48^b^
Protein (g L^−1^)		31.70 ± 0.01^a^	31.70 ± 0.05^a^	31.60 ± 0.01^a^	31.60 ± 0.05^a^	31.50 ± 0.02^a^
Viscosity (Cp)		2.16^a^	10.58^b^	11.31^c^	17.61^d^	29.73^e^
Fatty (g L^−1^)		33.4 ± 1.2^a^	33.33 ± 0.10^a^	32.33 ± 0.28^a^	33.43 ± 0.10^a^	33.33 ± 0.10^a^
Water activity a_w_		0.9725 ± 0.0060^a^	0.968 ± 0.004^a^	0.9605 ± 0.0030^b^	0.945 ± 0.002^c^	0.935 ± 0.001^d^
Colors	L*	65,293 ± 0.015^a^	72.72 ± 0.01^b^	70.11 ± 0.01^b^	67.78 ± 2.39^a^	73.75 ± 0.01^b^
	a*	−0.02 ± 0.01^a^	−0.08 ± 0.01^a^	−0.02 ± 0.01^a^	−0.24 ± 0.03^b^	−0.27 ± 0.01^b^
	b*	7.74 ± 0.01^a^	11.27 ± 0.01^b^	9.99 ± 0.01^c^	9.51 ± 0.05^c^	10.02 ± 0.01^bc^
	*∆E*	11.97 ± 0.01^a^	12.84 ± 0.01^b^	13.35 ± 0.01^b^	12.22 ± 0.01^ab^	12.30 ± 0.06^ab^
	C*	7.74 ± 0.01^a^	11.27 ± 0.01^b^	9.99 ± 0.01^c^	9.51 ± 0.05^c^	10.02 ± 0.01^bc^
Minerals	Na (mg L^−1^)	559.82 ± 29.83^a^	590.37 ± 8,97^ab^	592.56 ± 16,84^ab^	620.26 ± 54,31^b^	685.57 ± 2.51^c^
	K (mg L^−1^)	2317.34 ± 82.08^bc^	2173.79 ± 300.52^ab^	2037.48 ± 57.64^a^	1985.29 ± 241.04^cd^	1974.96 ± 127.46^d^
	Ca (mg L^1^)	1863.18 ± 107.62^a^	1641.97 ± 254.71^b^	1638.30 ± 47.01^b^	1586.56 ± 126.36^bc^	1547.03 ± 83.36^c^
	Mg (mg L^−1^)	102.54 ± 7.82^a^	109.35 ± 19.02^ab^	110.13 ± 0.17^ab^	116.70 ± 13.21^bc^	121.05 ± 9.11^c^
	Fe (mg L^−1^)	2.04 ± 0.36^a^	2.27 ± 0.17^a^	2.47 ± 1.22^a^	3.00 ± 0.84^a^	3.05 ± 0.42^a^

Values are mean ± SD (*n* = 3). Different letters in the same row indicate significant differences (*p* < 0.05). Asterisk symbol (*) denotes that the measurements were recorded using the standard CIE L*a*b* (1976) color space, as defined by the Commission Internationale de l’Éclairage.

The pH decreased significantly from 6.84 ± 0.35 at T0 to 4.44 ± 0.22 at T72 (*p* < 0.05), while titratable acidity increased from 19.35 ± 0.97 °D to 96.30 ± 0.48 °D (*p* < 0.05) (Figure 2). Dry matter showed a slight but significant decrease, from 122.86 ± 0.17 g L^−1^ at T0 to 120.67 ± 0.37 g L^−1^ at T72 (*p* < 0.05). Water activity (a__w_) significantly decreased from 0.9725 ± 0.0060 to 0.9350 ± 0.0010 (*p* < 0.05), while viscosity increased markedly from 2.16 cP at T0 to 29.73 cP at T72 (*p* < 0.05).

Protein and total fat contents remained relatively stable throughout fermentation, at an average of approximately 31.6 g L^−1^ and 33.4 g L^−1^, respectively (*p* > 0.05). Ash content decreased slightly from 6.38 ± 0.18 g L^−1^ to 6.01 ± 0.82 g L^−1^ (*p* < 0.05).

Mineral composition showed contrasting evolutions during fermentation. Sodium and magnesium concentrations increased significantly, from 559.82 ± 29.83 to 685.57 ± 2.51 mg L^−1^ and from 102.54 ± 7.82 to 121.05 ± 9.11 mg L^−1^, respectively (*p* < 0.05). In contrast, potassium and calcium concentrations decreased significantly, from 2317.34 ± 82.08 to 1974.96 ± 127.46 mg L^−1^ and from 1863.18 ± 107.62 to 1547.03 ± 83.36 mg L^−1^, respectively (*p* < 0.05). Iron concentration remained statistically unchanged throughout the fermentation period, ranging from 2.04 ± 0.36 to 3.05 ± 0.42 mg L^−1^ (*p* > 0.05).

Color parameters also showed significant changes. The lightness index (L*) varied between 65.29 ± 0.02 and 73.75 ± 0.01, while the redness index (a*) decreased from −0.02 ± 0.01 to −0.27 ± 0.01 (*p* < 0.05). The yellowness index (b*) increased significantly from 7.74 ± 0.01 at T0 to 10.02 ± 0.01 at T72 (*p* < 0.05). Total color difference (ΔE) and chroma (C*) also showed significant variations across fermentation time points (*p* < 0.05).

### 3.2 Organic acids and sugars

Marked changes in carbohydrate and organic acid concentrations were observed during the 72 h spontaneous fermentation (Table 2).

**Table 2:** Evolution of organic acid and sugar content in dromedary camel milk during 72 h of fermentation.

Organic acids	Fermentation time in hours				
	T0	T12	T24	T48	T72
Oxalate (mg L^−1^)	11,242	10,051	9,325	8,217	7,859
Citrate (mg L^−1^)	620,406	688,742	749,068	758.7	788,751
Quinic (mg L^−1^)	30,164	22,727	27,142	31,673	33,375
Lactate (mg L^−1^)	68,558	387,49	1,572,339	3,091,472	4,274,848
Formate (mg L^−1^)	135,809	189.2	245.6	312.4	178.9
Acetate (mg L^−1^)	246,107	180.5	95.2	42.1	ND
Succinate (mg L^−1^)	ND	193,068	319,209	319,723	376,192
Glucose (g L^−1^)	5.505	4.864	4.847	4.755	4.402
Lactose (g L^−1^)	49.380	37.120	23.280	11.846	5.254

Values are means ± SD (*n* = 3). Different superscript letters within the same row indicate significant difference (*p* < 0.05) according to Tukey’s *post-hoc* test.

ND, Not Detected.

Lactose concentration decreased significantly from 49.38 g L^−1^ at T0 to 37.12 g L^−1^ at T12, 23.28 g L^−1^ at T24, 11.85 g L^−1^ at T48, and 5.25 g L^−1^ at T72 (*p* < 0.05). The most pronounced decrease in lactose occurred between T0 and T24. Glucose concentration showed a smaller but significant decline, from 5.51 g L^−1^ at T0 to 4.40 g L^−1^ at T72 (*p* < 0.05).

Lactate concentration increased significantly throughout fermentation, from 68.56 mg L^−1^ at T0 to 387.49 mg L^−1^ at T12, 1572.34 mg L^−1^ at T24, 3091.47 mg L^−1^ at T48, and reached 4274.85 mg L^−1^ at T72 (*p* < 0.05). Succinate was not detected at T0, but appeared at T12 (193.07 mg L^−1^) and progressively increased to 376.19 mg L^−1^ at T72.

Formate concentration increased from 135.81 mg L^−1^ at T0 to a maximum of 312.40 mg L^−1^ at T48, then decreased to 178.90 mg L^−1^ at T72. Acetate concentration decreased steadily from 246.11 mg L^−1^ at T0 and became undetectable at T72. Citrate concentration increased progressively from 620.41 mg L^−1^ at T0 to 788.75 mg L^−1^ at T72 (*p* < 0.05). Oxalate showed a moderate but significant decrease from 11.24 to 7.86 mg L^−1^ (*p* < 0.05). Quinic acid concentration decreased from 30.16 mg L^−1^ at T0 to 22.73 mg L^−1^ at T12, then increased to 33.38 mg L^−1^ at T72.

### 3.3 Volatile compound profile

The evolution of volatile organic compounds (VOCs) during the spontaneous fermentation of Tunisian dromedary camel milk was monitored by VASE™-GC–MS (Figure 1). Eight volatile compounds were identified across the fermentation time points: ethanol, acetone, acetic acid, 2,3-butanedione (diacetyl), 2-butanone, acetoin, 2,3-butanediol, and hexanal. All observed variations in VOC intensities across fermentation time points were statistically significant (*p* < 0.05).

At T0, acetone and acetic acid exhibited the highest log₁₀ intensities (log₁₀ ≈ 8.1 and 7.2, respectively), while ethanol, acetoin, and 2,3-butanediol were detected at very low intensities (log₁₀ ≈ 5.9). After 12 h of fermentation, the intensities of ethanol, acetoin, and 2,3-butanediol increased markedly, reaching log₁₀ ≈ 8.8, 8.0, and 7.6, respectively.

Diacetyl was detected at low intensities at T0 and T12 (log₁₀ ≈ 5.9), then progressively increased from T24 onwards and reached its maximum intensity at T72 (log₁₀ ≈ 6.75). Between T24 and T48, ethanol (log₁₀ ≈ 8.5–8.7), acetoin (log₁₀ ≈ 7.8–8.0), and 2,3-butanediol (log₁₀ ≈ 7.4–7.5) remained the predominant volatile compounds. The intensity of acetone gradually declined throughout fermentation, from log₁₀ ≈ 8.1 at T0 to log₁₀ ≈ 7.6 at T72.

Hexanal was not detected during the first 24 h but appeared at T48 (log₁₀ ≈ 6.6) and persisted at T72 (log₁₀ ≈ 6.9). 2-Butanone remained at relatively low and stable intensities throughout fermentation (log₁₀ ≈ 5.9–6.3).

### 3.4 Fatty acid profile

The fatty acid composition of Tunisian dromedary camel milk during the 72 h spontaneous fermentation is presented in Table 3.

**Table 3:** Fatty acid composition (SFA%, MUFA%, PUFA%, USFA%) of dromedary camel milk during 72 h of fermentation.

Fatty acid profile	Fermentation time in hours				
	T0	T12	T24	T48	T72
SFA (%)	62.58	67.39	71.50	72.05	73.53
MUFA (%)	36.07	31.3	27.22	26.38	24.96
PUFA (%)	1.35	1.31	1.28	1.57	1.51
USFA (%)	37.42	32.61	28.50	27.95	26.47

The proportion of saturated fatty acids (SFA) increased significantly from 62.58% at T0 to 67.39% at T12, 71.50% at T24, 72.05% at T48, and 73.53% at T72 (*p* < 0.05). The proportion of total unsaturated fatty acids (USFA), including monounsaturated and polyunsaturated fatty acids, decreased correspondingly, from 37.42% at T0 to 26.47% at T72 (*p* < 0.05). The monounsaturated fatty acid (MUFA) fraction decreased significantly from 36.07 to 24.96% (*p* < 0.05), while the polyunsaturated fatty acid (PUFA) fraction remained relatively low throughout fermentation, ranging from 1.28 to 1.57%.

Despite these proportional changes in fatty acid distribution, the total fat content of the fermented milk remained statistically unchanged at approximately 33 g·L^−1^ throughout the 72 h fermentation period (Table 1).

### 3.5 Lactic acid bacteria growth

**Figure 3:**
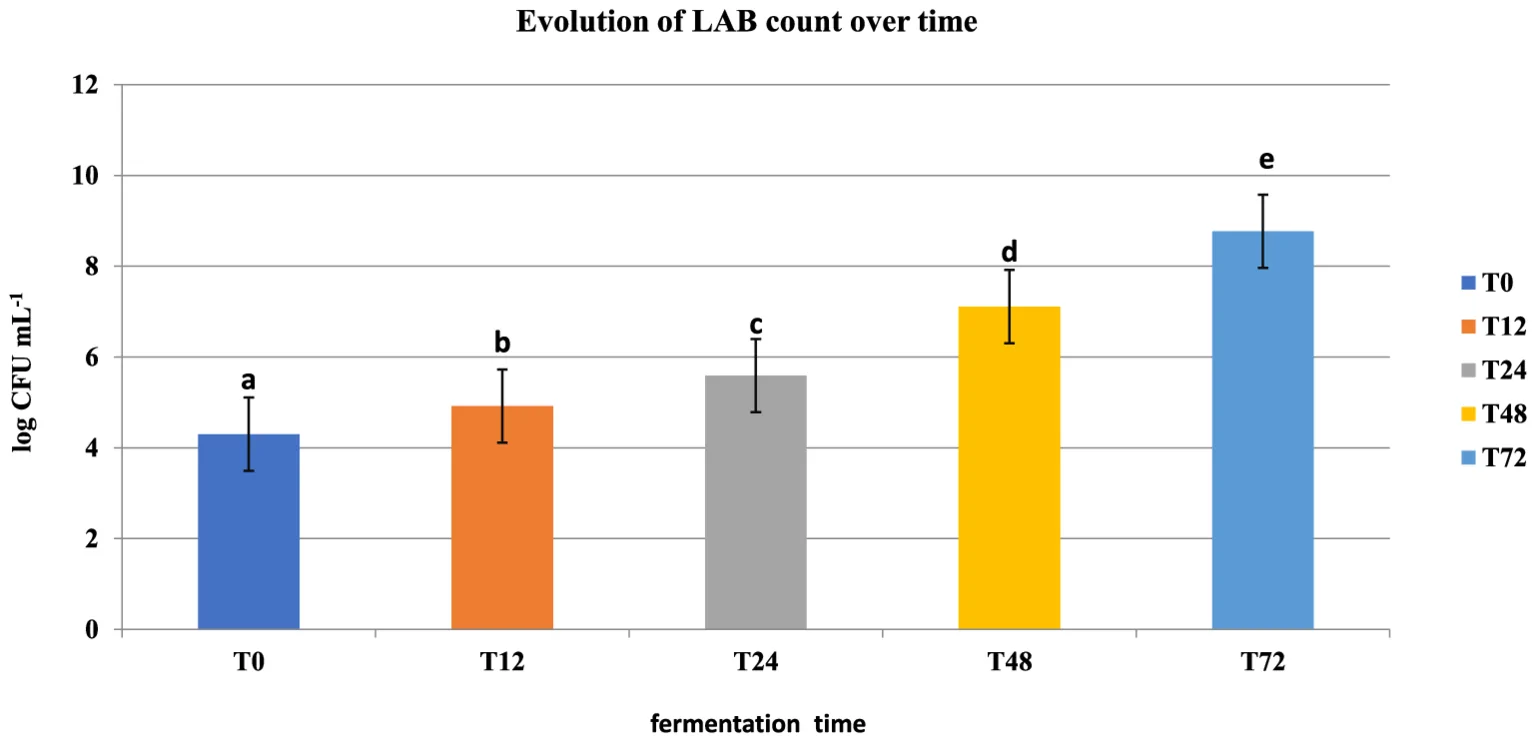
Evolution of lactic acid bacteria counts during spontaneous fermentation of dromedary camel milk. Different letters in the same row indicate significant differences (*p* < 0.05).

The evolution of LAB counts during the 72 h spontaneous fermentation of Tunisian dromedary camel milk is shown in Figure 3.

LAB counts increased significantly throughout fermentation, from 4.30 ± 0.02 log CFU·mL^−1^ at T0 to 4.95 ± 0.85 log CFU·mL^−1^ at T12, 5.60 ± 0.91 log CFU·mL^−1^ at T24, 7.10 ± 0.85 log CFU mL^−1^ at T48, and reached a maximum of 8.77 ± 0.80 log CFU·mL^−1^ at T72 (*p* < 0.05). This corresponds to an overall increase of approximately 4.5 log units over the 72 h fermentation period. The most pronounced increase in LAB counts was observed between T24 and T72.

### 3.6 Antioxidant activities

**Figure 4:**
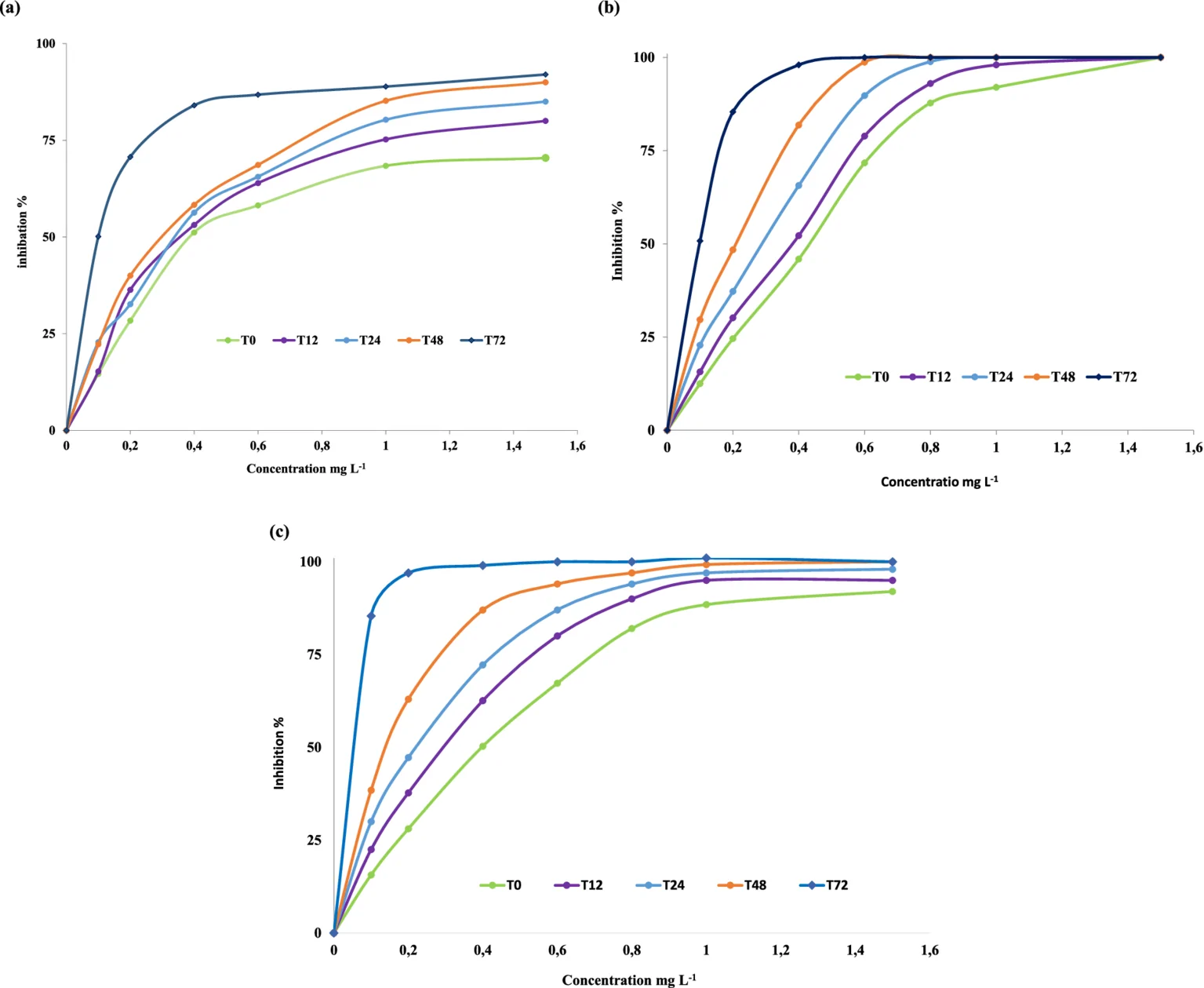
Chelating activity **(a)**, ABTS radical scavenging activity **(b)**, ß carotene antioxidant activity **(c)** in dromedary camel milk, expressed as percentage inhibition at different concentrations (mg mL^−1^).

The antioxidant activities of Tunisian dromedary camel milk during the 72 h spontaneous fermentation were evaluated using three complementary assays: ferrous ion chelating activity, ABTS radical-scavenging activity, and *β*-carotene bleaching inhibition (Figure 4). All three assays were performed at seven sample concentrations (0.1, 0.2, 0.4, 0.6, 0.8, 1.0, and 1.5 mg mL^−1^), and the IC₅₀ values calculated from the corresponding dose–response curves are summarized in Table 4.

**Table 4:** IC50 values of antioxidant activities of Tunisian dromedary camel milk during 72 h of fermentation, evaluated by three complementary assays: ferrous ion chelating activity (FCA), ABTS radical-scavenging activity, and B-carotene bleaching test (BCBT).

Antioxidant assay	T0	T12	T24	T48	T72
FCA–IC50 (mg mL^−1^)	0.55 ± 0.03^a^	0.45 ± 0.02^b^	0.35 ± 0.02^c^	0.28 ± 0.02^d^	0.20 ± 0.01^e^
ABTS–IC50 (mg ml^−1^)	0.50 ± 0.02^a^	0.40 ± 0.02^b^	0.32 ± 0.02^c^	0.25 ± 0.01^d^	0.18 ± 0.01^e^
BCBT – IC50 (mg ml^−1^)	0.32 ± 0.02^a^	0.22 ± 0.02^b^	0.15 ± 0.01^c^	0.10 ± 0.01^d^	0.08 ± 0.01^e^

Values are means ± SD (*n* = 3). Different superscript letters within the same row indicate significant difference (*p* < 0.05) according to Tukey’s *post-hoc* test.

For the ferrous ion chelating assay (Figure 4a), the inhibition percentage increased with both sample concentration and fermentation time. The IC₅₀ values decreased significantly from 0.55 ± 0.02 mg mL^−1^ at T0 to 0.20 ± 0.01 mg mL^−1^ at T72 (*p* < 0.05) (Table 4).

For the ABTS radical scavenging assay (Figure 4b), the dose–response curves shifted upward and leftward with increasing fermentation time. The IC₅₀ values decreased significantly from 0.50 ± 0.03 mg mL^−1^ at T0 to 0.18 ± 0.01 mg mL^−1^ at T72 (*p* < 0.05) (Table 4).

For the β-carotene bleaching assay (Figure 4c), inhibition percentages increased with both sample concentration and fermentation time. The IC₅₀ values decreased markedly from 0.45 ± 0.02 mg mL^−1^ at T0 to 0.08 ± 0.01 mg mL^−1^ at T72 (*p* < 0.05) (Table 4).

### 3.7 Antimicrobial activity

The antimicrobial activity of Tunisian dromedary camel milk during the 72 h spontaneous fermentation against four foodborne pathogens is presented in Table 5. All antimicrobial assays were performed after pH neutralization (pH 7.0 ± 0.1) and filtration (0.22 μm) of the fermented milk supernatants, in order to exclude acidity as the sole inhibitory factor. No inhibition zone was observed in wells containing sterile distilled water (negative control) in any of the assays.

**Table 5:** Antimicrobial activity of fermented dromedary camel milk against foodborne pathogens during 72 h of fermentation (inhibition zone diameter, mm).

Strain	T0 h	T12 h	T24 h	T48 h	T72 h
*Escherichia coli* ATCC 25922	13.7 ± 1.0^a^	16.8 ± 1.2^b^	18.5 ± 1.0^c^	21.3 ± 0.8^d^	22.0 ± 0.5^d^
*Staphylococcus aureus* ATCC 25923	11.3 ± 1.3^a^	14.5 ± 1.5^b^	18.0 ± 1.8^c^	19.3 ± 1.2^d^	21.5 ± 1.0^e^
*Bacillus cereus* ATCC 11778	6.7 ± 0.3^a^	15.5 ± 1.0^b^	17.3 ± 1.3^c^	18.5 ± 1.0^cd^	19.3 ± 0.7^d^
*Salmonella Typhi* ATCC 19430	9.3 ± 0.3^a^	9.5 ± 1.1^a^	12.5 ± 1.4^b^	13.5 ± 1.6^bc^	14.0 ± 1.2^c^
Negative control (sterile distilled water)	0.0 ± 0.0	0.0 ± 0.0	0.0 ± 0.0	0.0 ± 0.0	0.0 ± 0.0

Values are means ± SD (*n* = 3). Different superscript letters within the same row indicate significant difference (*p* < 0.05) according to Tukey’s *post-hoc* test.

Inhibition zone diameters increased significantly from T0 to T72 against all four pathogens tested (*p* < 0.05). For *Escherichia coli* ATCC 25922, inhibition zone diameter increased from 13.7 ± 0.6 mm at T0 to 22.0 ± 0.5 mm at T72. For *Staphylococcus aureus* ATCC 25923, inhibition zone diameter increased from 11.3 ± 1.3 mm to 21.5 ± 1.0 mm. For *Bacillus cereus* ATCC 11778, inhibition zone diameter increased from 6.7 ± 0.3 mm to 19.3 ± 0.7 mm. For *Salmonella Typhi* ATCC 19430, inhibition zone diameter increased from 9.3 ± 0.3 mm to 14.0 ± 1.2 mm.

The largest relative increase in inhibition zone diameter over the 72 h fermentation was observed for *Bacillus cereus* (+188%), followed by *Staphylococcus aureus* (+90%), *Escherichia coli* (+60%), and *Salmonella Typhi* (+51%). Even at T0, raw camel milk exhibited measurable antimicrobial activity against all four pathogens, with inhibition zone diameters ranging from 6.7 ± 0.3 mm to 13.7 ± 0.6 mm.

## 4 Discussion

It should be noted that, since microbial characterization in the present study was limited to culture-dependent enumeration on selective media, the mechanistic interpretations proposed in the following sections are presented as plausible explanations supported by previous literature on LAB and fermented dairy systems, rather than as mechanisms experimentally demonstrated within this work. The specific contributions of individual microbial taxa, enzymatic pathways, and bioactive compounds remain to be confirmed by future molecular, biochemical, and peptidomic investigations.

### 4.1 Physicochemical and matrix transformations during fermentation

The spontaneous fermentation of dromedary camel milk over 72 h brought about substantial biochemical and structural transformations that progressively enriched the milk with functional properties beyond those observed in the raw material. The acidification dynamics, as indicated by the decline in pH and the corresponding rise in titratable acidity, are consistent with the metabolic activity of indigenous LAB. These trends are in agreement with previous reports on spontaneous camel milk fermentation and may be primarily attributed to lactose conversion into lactic acid (35). It is noteworthy that metabolic activity was prolonged beyond the stationary phase of LAB growth, possibly owing to acid-resistant strains that can maintain enzymatic activity in increasingly adverse conditions (22, 36).

The slight decrease in dry matter (from 122.86 to 120.67 g L^−1^) may reflect the microbial consumption of lactose, while the decline in water activity (from 0.9725 to 0.9350) suggests a higher concentration of dissolved metabolites. Although these changes are modest, they may contribute to the hurdle technology effect, reinforcing the stability of the fermented camel milk alongside acidification. The constancy of protein (~31.6 g L^−1^) and fat (~33.4 g L^−1^) contents throughout fermentation is encouraging from a nutritional standpoint, as it indicates that the macronutritional composition of the final product remains similar to that of raw camel milk, despite molecular-level alterations (24, 37).

Another interesting phenomenon is the dynamics of minerals during fermentation. The sharp decrease in potassium and calcium levels, accompanied by a corresponding increase in magnesium and sodium, is consistent with a redistribution of minerals from the colloidal to the soluble phase, primarily due to the solubilization of calcium phosphate under acidic conditions (4). Compared to bovine casein micelles, camel milk caseins display different interactions with minerals and lack *β*-lactoglobulin, which may result in different aggregation patterns under acidic conditions (38). These mineral changes are likely related to the structuration of the gel, as supported by the increase in viscosity values (from 2.16 to 29.73 cP) and color changes such as a rise in yellowness index (b*), possibly due to colloidal changes and incipient Maillard-type reactions (39, 40).

A distinctive feature of the mineral profile evolution was the complete retention of iron (Fe) throughout the 72 h fermentation period. Whereas significant declines were recorded for calcium and potassium, iron concentrations remained statistically unchanged (*p* > 0.05). The initial concentration (2.04 mg L^−1^) is consistent with reported values for dromedary milk (13), and its stability under acidic conditions (pH 4.44) is noteworthy. The most plausible explanation lies in the unique protein composition of camel milk. Camel milk is exceptionally rich in lactoferrin, an iron-binding glycoprotein present at concentrations significantly higher than in bovine milk (41). Lactoferrin exhibits a high affinity for ferric ions (Fe^3+^) and remains structurally stable at the pH values and fermentation duration tested here (10). The limited proteolysis observed (stable total protein values) suggests that the lactoferrin-iron complex remained intact, preventing iron from precipitating as insoluble hydroxides or phosphates. From a nutritional standpoint, the preservation of iron content is of considerable importance. Iron deficiency anemia is prevalent in arid regions where camel milk is a dietary staple. The fact that spontaneous fermentation does not diminish this content reinforces the value of traditionally fermented dromedary camel milk as a functional food capable of contributing to micronutrient intake (2, 4).

### 4.2 Organic acid and sugar metabolism

The progressive decrease in lactose concentration, accompanied by the marked accumulation of lactic acid, is consistent with active carbohydrate metabolism by indigenous LAB. However, since the microbial community was not characterized at the genus or species level, the specific microbial pathways involved cannot be directly assigned and are interpreted here in light of mechanisms previously reported in similar fermentation systems (19, 20). The relatively limited reduction in glucose levels over the fermentation period suggests that glucose metabolism might have been favored in the initial stages of fermentation prior to lactose becoming a major energy source (22).

The marked increase in lactic acid concentration after 72 h identifies it as a major fermentation end product. However, lactic acid was not the only organic acid produced. The later appearance of succinate, the initial increase and subsequent reduction in formate levels, the continuous reduction in acetate levels, and the later increase in citrate and quinic acid levels all point to a diversification of fermentation processes beyond typical lactic acid fermentation. These secondary fermentation processes may become more significant in later stages of fermentation as readily available energy sources are consumed and as the microbial culture adapts to the acidic environment (20). The organic acids may also contribute not only to preservation but also to the taste of the fermented product (42).

### 4.3 Volatile profile

The kinetic study suggests that the spontaneous fermentation of Tunisian dromedary milk proceeds through distinct metabolic stages, each characterized by a specific volatile organic compound (VOC) profile.

#### 4.3.1 Early metabolic activity (0–12 h): possible involvement of citrate-related metabolism

The rapid emergence of ethanol, acetoin, and 2,3-butanediol within the first 12 h is consistent with the early establishment of active LAB populations, as commonly described in spontaneously fermented dairy systems (15, 23). The sequential evolution of the volatile profile is in agreement with the metabolic kinetics previously reported for the citrate/pyruvate pathway in LAB (43, 44). According to these previous studies, *α*-acetolactate derived from citrate-sourced pyruvate may be enzymatically converted to acetoin by acetolactate decarboxylase, and acetoin may be further reduced to 2,3-butanediol by NADH-dependent butanediol dehydrogenase. Diacetyl, in turn, has been reported to result from non-enzymatic oxidative decarboxylation of α-acetolactate, a process requiring oxygen and a sufficient precursor pool (45), which could explain its delayed appearance at 24 h. The transient peak of acetic acid observed at 12 h is also consistent with citrate cleavage, which has been reported to stoichiometrically release one acetate molecule (46). As the indigenous microbiota was not characterized at the molecular level in the present study, these pathways are proposed as plausible explanations supported by previous literature, rather than as mechanisms experimentally demonstrated in this work.

#### 4.3.2 Intermediate phase (12–48 h): ketone shifts

Acetone, which was the dominant volatile compound in raw milk (log₁₀ ≈ 8.1 at T0), declined steadily after 12 h. This decrease may be related to its potential enzymatic reduction to 2-propanol by secondary alcohol dehydrogenases (sADH) reported in various microbial systems (47). According to previous studies, sADH activity has been associated with the maintenance of intracellular redox balance during microbial growth. However, the specific microbial origin of this activity in spontaneously fermented Tunisian dromedary milk cannot be confirmed without molecular identification of the indigenous microbiota and remains to be investigated.

#### 4.3.3 Late phase (48–72 h): possible re-oxidation and lipid degradation

Between 48 and 72 h, a progressive decline in 2,3-butanediol was observed concurrently with an increase in acetoin and diacetyl. This pattern is consistent with a possible partial re-oxidation of 2,3-butanediol to acetoin, a reaction that has been previously associated with the reverse action of butanediol dehydrogenase under conditions of increased oxygen availability and altered NADH/NAD^+^ balance (48, 49). The late appearance of hexanal from T48 onwards is also consistent with the onset of lipid oxidation, particularly the breakdown of *ω*-6 polyunsaturated fatty acids such as linoleic acid, which has been widely reported as a marker of this process (50). This trend may be related to the progressive acidification of the matrix and the potential destabilization of fat globule membranes, which could expose unsaturated fatty acids to residual oxygen. However, these mechanisms are proposed as plausible explanations based on previous literature and were not directly investigated in the present study.

### 4.4 Fatty acid profile

The lipid fraction was found to possess considerable stability throughout the entire fermentation process, where SFA consistently dominated and even increased from 62.58 to 73.53% of total fatty acids. This stability, along with the constant level of total fat content, points to a low level of lipolytic activity in the entire process, although the increase in SFA could be related to the activity of the native microflora (3, 4, 51, 52).

### 4.5 LAB growth

Throughout the 72 h incubation period, the LAB population showed a marked growth rate, with counts increasing from the initial log 4.3 CFU mL^−1^ to the range of log 8–9 CFU mL^−1^. The temporal correspondence between the peak growth of LAB and the maximum rate of pH decrease supports the central role of LAB in the acidification process (20). Based on the literature on spontaneous camel milk fermentation, the indigenous microflora likely included genera such as *Lactobacillus*, *Lactococcus, Leuconostoc*, and *Weissella*, some of which have been reported to exhibit probiotic attributes in camel milk systems (15, 17, 22).

### 4.6 Antioxidant activities

The three complementary antioxidant assays performed in the present study consistently revealed a progressive enhancement of the antioxidant capacity of Tunisian dromedary camel milk during the 72 h spontaneous fermentation (Figure 4 and Table 4). The IC₅₀ values decreased significantly for the ferrous ion chelating assay (from 0.55 to 0.20 mg mL^−1^), the ABTS radical-scavenging assay (from 0.50 to 0.18 mg mL^−1^), and the *β*-carotene bleaching assay (from 0.45 to 0.08 mg mL^−1^) (*p* < 0.05). This convergent improvement across three mechanistically distinct assays suggests that fermentation promoted a multi-mechanistic antioxidant response, involving the sequestration of pro-oxidant transition metals, the scavenging of free radicals, and the inhibition of lipid peroxidation.

A plausible explanation for this enhancement is the potential proteolytic activity of indigenous LAB on caseins and whey proteins, which has been previously reported to release peptides enriched in hydrophobic and aromatic amino acids with antioxidant properties (36). Such peptides have been described as capable of chelating pro-oxidant metal ions, donating electrons or hydrogen atoms to neutralize free radicals, and stabilizing oil-in-water emulsions, consistent with the convergent improvement observed across the three assays (36, 42, 53). The high native lactoferrin content of dromedary milk (10, 41) and its potential enzymatic hydrolysis by indigenous LAB may further contribute to the metal-chelating activity by releasing smaller iron-binding peptides (10, 53). Notably, the IC₅₀ values reached at T72 in the present study are comparable to or lower than values previously reported for fermented camel milk obtained with selected LAB starters (54–56), suggesting that the indigenous microbiota of Tunisian dromedary milk possesses a comparable or enhanced potential to generate antioxidant compounds.

However, since the proteolytic profile, peptide composition, and microbial community of the fermented milk were not directly characterized in the present study, these interpretations are presented as plausible explanations supported by the literature and should be confirmed by future peptidomic and microbial analyses. Overall, the convergent enhancement of antioxidant activity across the three complementary assays supports the value of spontaneously fermented dromedary milk as a potential source of natural antioxidants, with relevance for the development of functional fermented dairy products.

### 4.7 Antimicrobial activity

The systematic inclusion of a sterile distilled water control, which consistently produced no measurable inhibition zone, supports the methodological reliability of the agar well diffusion assay. This observation is consistent with the interpretation that the antimicrobial effects reported in the present study originated specifically from the bioactive compounds contained in the fermented camel milk supernatants, rather than from artefacts of the diffusion procedure.

The progressive increase in the inhibition zone diameters against *Escherichia coli*, *Staphylococcus aureus*, *Bacillus cereus*, and *Salmonella Typhi* during the fermentation process suggests that the antimicrobial activity of camel milk is significantly enhanced during spontaneous fermentation. The progressive increase in lactic acid concentration over fermentation time can be associated with the observed improvement in antimicrobial activity, further supporting the role of organic acids in pathogen inhibition. However, since the assays were performed after pH neutralization, the observed antimicrobial activity cannot be attributed solely to lactic acid, suggesting the contribution of additional inhibitory compounds.

The observed antimicrobial activity may therefore be attributed to the combined contribution of several factors previously described in fermented dairy systems, including organic acids, hydrogen peroxide, potential bacteriocins produced by indigenous LAB, and endogenous antimicrobial proteins of camel milk such as lactoferrin and lysozyme, which have been reported to be more abundant in camel milk than in bovine milk (5, 41). Since the LAB community was not characterized at the molecular level in the present study, the production of specific bacteriocins or other strain-dependent antimicrobial compounds could not be directly demonstrated and is proposed here as a plausible mechanism supported by previous literature.

This sustained antimicrobial activity assumes particular importance, especially in arid and hot climates where refrigeration facilities are limited. The potential involvement of bacteriocins produced by indigenous LAB strains has been described in similar systems and may contribute to selective antimicrobial activity against specific pathogens (5). However, confirming this contribution would require molecular identification of the indigenous LAB community and functional characterization of their antimicrobial metabolites in future studies.

### 4.8 Nutritional implications and functional food perspective

Overall, the findings of this research support the trend towards the recognition of fermented dromedary camel milk as a functional food with considerable nutritional and therapeutic value. The decrease in lactose content may make the milk more appealing to lactose-intolerant consumers, while the improvement in antioxidant and antimicrobial properties suggests potential health-promoting properties, including antioxidant protection and immune modulation (57, 58). However, since the bioactive compounds responsible for these effects were not directly identified in the present study, the physiological relevance of these functional properties should be confirmed by future biochemical, peptidomic, and *in vivo* investigations. Instead of focusing on the chemical structure of specific bioactive compounds, the current research attempted to trace the time course of the development of the enhanced properties of the milk during spontaneous fermentation, thus taking a holistic approach toward understanding the traditional process of transforming raw camel milk into a more nutritious and therapeutically valuable product. This is particularly relevant in arid and semi-arid regions where camel milk represents an important dietary resource and where the traditional practice of spontaneous fermentation contributes to both food security and functional food development.

### 4.9 Limitations and future directions

Several limitations of the present study should be acknowledged.

First, microbial characterization was limited to culture-dependent enumeration of LAB on selective media, without molecular identification at the genus, species, or strain level. As a result, the specific microbial taxa responsible for the observed metabolic, biochemical, and functional changes during spontaneous fermentation could not be directly identified, and the mechanistic interpretations proposed in this work are presented as plausible explanations supported by the literature rather than as experimentally demonstrated mechanisms (16, 17, 20). Future studies should include culture-independent approaches, such as 16S rRNA gene sequencing or shotgun metagenomics, to characterize the indigenous microbial communities of Tunisian dromedary milk.

Second, although the antioxidant and antimicrobial activities of the fermented milk were significantly enhanced during fermentation, the bioactive compounds underlying these effects were not directly identified. The release of bioactive peptides, in particular, was inferred from the observed activities and from previously published mechanisms but was not experimentally demonstrated. Future work should include targeted peptidomic and proteomic analyses to identify and characterize the bioactive peptides generated during the spontaneous fermentation of camel milk.

Third, spontaneous fermentation is inherently associated with variability arising from geographic origin, seasonal effects, animal feeding regimes, and the composition of the indigenous microbiota. The use of three independent biological replicates partially addresses this variability and supports the reproducibility of the kinetic patterns reported here. However, broader sampling across regions and seasons would further strengthen the generalizability of the findings.

Fourth, although the volatile organic compounds were identified using a robust dual criterion approach (NIST library matching combined with retention index validation using authentic pure standards), the quantification remained semi-quantitative, based on log₁₀-transformed peak areas rather than absolute concentrations. Future studies could benefit from targeted quantification using internal standards or isotope-labeled compounds to provide absolute concentration data for the volatile compounds generated during spontaneous fermentation of Tunisian dromedary milk.

Fifth, while one-way ANOVA followed by Tukey’s HSD test was used to compare parameter values across fermentation time points, formal verification of ANOVA assumptions (normality and homogeneity of variances) was not performed in the present study. The choice of parametric tests was justified by the relatively low variability observed within biological replicates and by the consistency of results across complementary parameters. Nevertheless, future studies should systematically include assumption-checking procedures (e.g., Shapiro–Wilk test for normality and Levene’s test for homogeneity of variances) and consider non-parametric alternatives when appropriate.

Finally, the physiological relevance of the functional properties reported here was not validated through *in vitro* digestion models or *in vivo* studies. Future research should address these aspects in order to translate the kinetic and biochemical findings of the present work into evidence-based health-related claims, in line with previous recommendations for fermented camel milk products (54, 59, 60).

## 5 Conclusion

The spontaneous fermentation of dromedary camel milk over 72 h resulted in significant changes in the physical, chemical, and biological characteristics of the milk. The decrease in pH and increase in titratable acidity are consistent with active lactic acid fermentation. Viscosity increased and water activity decreased, suggesting the formation of a more structured gel matrix with improved stability. Concurrently, LAB proliferation and changes in the organic acid and sugar contents were dramatic, with extensive breakdown of lactose and accumulation of lactic and succinic acids.

The significant changes in the fermented product were associated with a marked increase in antioxidant activity, as evidenced by enhanced metal-chelating, ABTS, and *β*-carotene bleaching assays. These observations are consistent with previous reports describing the potential contribution of bioactive peptides and other low-molecular-weight compounds generated during dromedary camel milk fermentation (36, 53, 55). However, direct identification of these compounds in the present study would require additional peptidomic and chemical characterization.

The fermented product also showed potent antimicrobial activity against major foodborne pathogens, with inhibition zone diameters increasing significantly from T0 to T72 for all four pathogens tested. Since the antimicrobial assays were performed after pH neutralization to pH 7.0 ± 0.1, the observed inhibitory activity cannot be attributed solely to acidification. The antimicrobial activity is therefore likely to result from the combined contribution of organic acids, bioactive peptides potentially released by indigenous LAB proteolytic activity, and endogenous antimicrobial proteins inherent to dromedary camel milk, such as lactoferrin and lysozyme, which have been reported to be more abundant in camel milk than in bovine milk (5, 41).

The results of this study suggest the potential for spontaneously fermented dromedary camel milk to serve as a functional food with health-promoting properties for oxidative stress prevention, gut health promotion, and food safety improvement.

Further research should focus on the molecular characterization of the indigenous microbiota responsible for spontaneous fermentation of Tunisian dromedary milk, the identification of the bioactive peptides and other compounds underlying the observed antioxidant and antimicrobial activities, and *in vitro* digestion and *in vivo* validation studies to confirm the physiological relevance of these functional properties. These complementary approaches will help substantiate the use of spontaneously fermented dromedary camel milk as a functional food.

## Data Availability

The original contributions presented in the study are included in the article/supplementary material, further inquiries can be directed to the corresponding authors.

## References

[1] Elhassan MMO Eissa L Elseory AMA Al-Thnaian TA. The dromedary camel as a climate-resilient species: unique digestive system morphology supporting life in harsh environments. Assiut Vet Med J. (2026) 72:160–74. doi: 10.21608/avmj.2026.401189.1786

[2] Arain MA Salman HM Ali M Khaskheli GB Barham GS Marghazani IB . A review on camel milk composition, techno-functional properties and processing constraints. Food Sci Anim Resour. (2024) 44:739–57. doi: 10.5851/kosfa.2023.e18, 38974725 PMC11222694

[3] Khaliq A Mishra AK Niroula A Baba WN Shaukat MN Rabbani A. An updated comprehensive review of camel milk: composition, therapeutic properties, and industrial applications. Food Biosci. (2024) 62:105531. doi: 10.1016/j.fbio.2024.105531

[4] Seifu E. Camel milk products: innovations, limitations and opportunities. Food Prod Process Nutr. (2023) 5:15. doi: 10.1186/s43014-023-00130-7

[5] Hamed NS Mbye M Ayyash M Ulusoy BH Kamal-Eldin A. Camel milk: antimicrobial agents, fermented products, and shelf life. Foods. (2024) 13:381. doi: 10.3390/foods13030381, 38338516 PMC10855775

[6] Mefleh M Darwish AMG Mudgil P Maqsood S Boukid F. Traditional fermented dairy products in southern Mediterranean countries: from tradition to innovation. Fermentation. (2022) 8:743. doi: 10.3390/fermentation8120743

[7] Roy D Ye A Moughan PJ Singh H. Composition, structure, and digestive dynamics of milk from different species-a review. Front Nutr. (2020) 7:577759. doi: 10.3389/fnut.2020.577759, 33123547 PMC7573072

[8] Ho TM Zou Z Bansal N. Camel milk: a review of its nutritional value, heat stability, and potential food products. Food Res Int. (2022) 153:110870. doi: 10.1016/j.foodres.2021.110870, 35227464

[9] Abd El-Aziz M Kassem JM Assem FM Abbas HM. Physicochemical properties and health benefits of camel milk and its applications in dairy products: a review. Egypt J Chem. (2022) 65:101–18. doi: 10.21608/ejchem.2021.92589.4383

[10] Al-Shamsi KA Mudgil P Hassan HM Maqsood S. Camel milk protein hydrolysates with improved techno-functional properties and enhanced antioxidant potential in in vitro and in food model systems. J Dairy Sci. (2018) 101:47–60. doi: 10.3168/jds.2017-13194, 29128226

[11] Mudgil P Redha AA Nirmal NP Maqsood S. In vitro antidiabetic and antihypercholesterolemic activities of camel milk protein hydrolysates derived upon simulated gastrointestinal digestion of milk from different camel breeds. J Dairy Sci. (2023) 106:3098–108. doi: 10.3168/jds.2022-22701, 36935238

[12] Ji Y Silva M Lin R Adhikari B Chandrapala J. Gelation and thermal stability of camel milk protein and soy protein blends: a review. Food Rev Int. (2024) 40:3816–46. doi: 10.1080/87559129.2024.2374810

[13] Konuspayeva G Faye B Gagaoua M Dib AL Bererhi E Pulina G. Recent advances in camel milk processing. Animals. (2021) 11:1045. doi: 10.3390/ani11041045 PMC8068116 33917722

[14] Benkerroum N Boughdadi A Bennani N Hidane K. Microbiological quality assessment of Moroccan camel's milk and identification of predominating lactic acid bacteria. World J Microbiol Biotechnol. (2003) 19:645–8. doi: 10.1023/A:1025114601811

[15] Belkheir K Laref N. Autochthonous lactic acid bacteria from *Camelus dromedarius* milk: an updated systematic review. Food Rev Int. (2025) 41:1576–91. doi: 10.1080/87559129.2025.2450266

[16] Rahmeh R Akbar A Alomirah H Kishk M Al-Ateeqi A Al-Milhm S . Camel milk microbiota: a culture-independent assessment. Food Res Int. (2022) 159:111629. doi: 10.1016/j.foodres.2022.111629, 35940813

[17] Bilal Z Akhmetsadykova S Baubekova A Tormo H Faye B Konuspayeva G. The main features and microbiota diversity of fermented camel milk. Foods. (2024) 13:1985. doi: 10.3390/foods13131985, 38998490 PMC11240983

[18] de Souza EL de Oliveira KÁ de Oliveira ME. Influence of lactic acid bacteria metabolites on physical and chemical food properties. Curr Opin Food Sci. (2023) 49:100981. doi: 10.1016/j.cofs.2022.100981

[19] Attia H Kherouatou N Dhouib A. Dromedary milk lactic acid fermentation: microbiological and rheological characteristics. J Ind Microbiol Biotechnol. (2001) 26:263–70. doi: 10.1038/sj.jim.7000111, 11494100

[20] Sun M Shao W Liu Z Ma X Chen H Zheng N . Microbial diversity in camel milk from Xinjiang, China as revealed by metataxonomic analysis. Front Microbiol. (2024) 15:1367116. doi: 10.3389/fmicb.2024.1367116, 38533337 PMC10964795

[21] Zhao J Fan H Kwok LY Guo F Ji R Ya M . Analyses of physicochemical properties, bacterial microbiota, and lactic acid bacteria of fresh camel milk collected in Inner Mongolia. J Dairy Sci. (2020) 103:106–16. doi: 10.3168/jds.2019-17023, 31629514

[22] Latreche B Bendjama E Loucif L Sanah I Messaoudi M Bensouici C . Unveiling the potential of lactic acid bacteria from Algerian dromedary camel milk: diversity, technological applications, and antimicrobial insights. Front Nutr. (2025) 12:1647344. doi: 10.3389/fnut.2025.1647344, 41049382 PMC12488446

[23] Alam MK Prete R Rannou C Lethuaut L Faieta M Perla C . Microbial impact on the volatilomic profile of fermented milks: a case study with the application of industrial starter cultures. Appl Food Res. (2025) 5:101254. doi: 10.1016/j.afres.2025.101254

[24] Zhou Y Wang D Zhao J Guo Y Yan W. Differentiation and characterization of volatile compounds in five common milk powders using HS-GC-IMS, HS-SPME-GC-MS, and multivariate statistical approaches. Food Chem X. (2025) 25:102179. doi: 10.1016/j.fochx.2025.102179, 39906067 PMC11791332

[25] Zhadyra S Tao F Xu P. Two-dimensional GC-TOFMS analysis of volatile organic compounds in fermented camel milk (Shubat). Foods. (2025) 14:2995. doi: 10.3390/foods14172995, 40941111 PMC12427637

[26] Psillakis E. Vacuum-assisted headspace solid-phase microextraction: a tutorial review. Anal Chim Acta. (2017) 986:12–24. doi: 10.1016/j.aca.2017.06.033, 28870316

[27] Vakinti M Mela SM Fernández E Psillakis E. Room temperature and sensitive determination of haloanisoles in wine using vacuum-assisted headspace solid-phase microextraction. J Chromatogr A. (2019) 1602:142–9. doi: 10.1016/j.chroma.2019.03.047, 30961964

[28] He X Yangming H Górska-Horczyczak E Wierzbicka A Jeleń HH. Rapid analysis of baijiu volatile compounds fingerprint for their aroma and regional origin authenticity assessment. Food Chem. (2021) 337:128002. doi: 10.1016/j.foodchem.2020.128002, 32927226

[29] Jeleń HH Gaca A Marcinkowska M. Use of sorbent-based vacuum extraction for determination of volatile phenols in beer. Food Anal Methods. (2018) 11:3089–94. doi: 10.1007/s12161-018-1277-z

[30] El Hatmi H Jrad Z Oussaief O Nasri W Sbissi I Khorchani T . Fermentation of dromedary camel (*Camelus dromedarius*) milk by *enterococcus faecium* and *Streptococcus macedonicus* as a potential alternative to fermented cow milk. LWT. (2018) 90:373–80. doi: 10.1016/j.lwt.2017.12.040

[31] Chan EWC Lim YY Chew YL. Antioxidant activity of *Camellia sinensis* leaves and tea from a lowland plantation in Malaysia. Food Chem. (2007) 102:1214–22. doi: 10.1016/j.foodchem.2006.07.009

[32] Re R Pellegrini N Proteggente A Pannala A Yang M Rice-Evans C. Antioxidant activity applying an improved ABTS radical cation decolorization assay. Free Radic Biol Med. (1999) 26:1231–7. doi: 10.1016/S0891-5849(98)00315-3, 10381194

[33] Koleva II Van Beek TA Linssen JPH De Groot A Evstatieva LN. Screening of plant extracts for antioxidant activity: a comparative study on three testing methods. Phytochem Anal. (2002) 13:8–17. doi: 10.1002/pca.611, 11899609

[34] Mather AE Gilmour MW Reid SWJ French NP. Foodborne bacterial pathogens: genome-based approaches for enduring and emerging threats in a complex and changing world. Nat Rev Microbiol. (2024) 22:543–55. doi: 10.1038/s41579-024-01051-z, 38789668

[35] Khosravi-Darani K Jahadi M Abbasi H Asgari M Tarlak F. Production of chocolate probiotic dessert based on camel milk using *Lacticaseibacillus casei*. Carpathian J Food Sci Technol. (2022) 14:189–206. doi: 10.34302/crpjfst/2022.14.2.16

[36] El-Sayed MI Awad S Abou-Soliman NHI. Improving the antioxidant properties of fermented camel milk using some strains of *Lactobacillus*. Food Nutr Sci. (2021) 12:352–71. doi: 10.4236/fns.2021.124028

[37] Warakaulle S Ayyash MM Kamal-Eldin A. Profiling protein hydrolysis and amino acid metabolism in camel and bovine milk fermented by *Lactobacillus helveticus*, *L. delbrueckii* subsp. *bulgaricus*, and *Streptococcus salivarius* subsp. *thermophilus*. Sci Rep. (2025) 15:18371. doi: 10.1038/s41598-025-02944-6 PMC12106841 40419683

[38] Ali SN Asim SM Samad N Ayyash M Kamal-Eldin A. Microstructural and rheological properties of camel and bovine milk fermented with five lactic acid bacteria strains. Foods. (2026) 15:546. doi: 10.3390/foods15030546, 41683133 PMC12896414

[39] Koirala P Dahal M Rai S Dhakal M Nirmal NP Maqsood S . Dairy milk protein–derived bioactive peptides: avengers against metabolic syndrome. Curr Nutr Rep. (2023) 12:308–26. doi: 10.1007/s13668-023-00472-1, 37204636 PMC10198026

[40] El-Hatmi H Oussaief O Hammadi I Dbara M Hammadi M Khorchani T . Relation between color and chemical composition of dromedary camel colostrum. Animals (Basel). (2023) 13:442. doi: 10.3390/ani13030442, 36766331 PMC9913735

[41] El-Hatmi H Girardet JM Gaillard JL Yahyaoui MH Attia H. Characterisation of whey proteins of camel (*Camelus dromedarius*) milk and colostrum. Small Rumin Res. (2007) 70:267–71. doi: 10.1016/j.smallrumres.2006.04.001

[42] Mahmoudi I Ben Moussa O Boulares M Chouaibi M Hassouna M. Novel probiotic camel milk yoghurt supplemented with inulin: antibacterial, antioxidant and antidiabetic effects. Mljekarstvo. (2022) 72:201–12. doi: 10.15567/mljekarstvo.2022.0402

[43] Hugenholtz J. Citrate metabolism in lactic acid bacteria. FEMS Microbiol Rev. (1993) 12:165–78. doi: 10.1111/j.1574-6976.1993.tb00017.x

[44] Pudlik AM Lolkema JS. Citrate uptake in exchange with intermediates in the citrate metabolic pathway in *Lactococcus lactis* IL1403. J Bacteriol. (2011) 193:706–14. doi: 10.1128/JB.01171-10, 21115655 PMC3021216

[45] Starrenburg MJC Hugenholtz J. Citrate fermentation by Lactococcus and Leuconostoc spp. Appl Environ Microbiol. (1991) 57:3535–40. doi: 10.1128/aem.57.12.3535-3540.1991, 16348602 PMC184008

[46] García-Quintáns N Repizo G Martín M Magni C López P. Activation of the diacetyl/acetoin pathway *in Lactococcus lactis subsp*. *lactis* bv. Diacetylactis CRL264 by acidic growth. Appl Environ Microbiol. (2008) 74:1988–96. doi: 10.1128/AEM.01851-07, 18245243 PMC2292580

[47] Sutak R Hrdý I Doležal P Cabala R Sedinová M Lewin J . Secondary alcohol dehydrogenase catalyzes the reduction of exogenous acetone to 2-propanol in *trichomonas vaginalis*. FEBS J. (2012) 279:2768–80. doi: 10.1111/j.1742-4658.2012.08661.x, 22686835

[48] Xiao Z Xu P. Acetoin metabolism in bacteria. Crit Rev Microbiol. (2007) 33:127–40. doi: 10.1080/10408410701364604 17558661

[49] Liu J Chan SHJ Brock-Nannestad T Chen J Lee SY Solem C . Combining metabolic engineering and biocompatible chemistry for high-yield production of homo-diacetyl and homo-(S,S)-2,3-butanediol. Metab Eng. (2016) 36:57–67. doi: 10.1016/j.ymben.2016.02.008, 26969254

[50] Frankel EN Neff WE Selke E. Analysis of autoxidized fats by gas chromatography-mass spectrometry. IX. Homolytic vs. heterolytic cleavage of primary and secondary oxidation products. Lipids. (1984) 19:790–800. doi: 10.1007/BF02534473

[51] Wang F Chen M Luo R Huang G Wu X Zheng N . Fatty acid profiles of milk from Holstein cows, Jersey cows, buffalos, yaks, humans, goats, camels, and donkeys based on gas chromatography–mass spectrometry. J Dairy Sci. (2022) 105:1687–700. doi: 10.3168/jds.2021-20750, 34802741

[52] Bakry IA Yang L Farag MA Korma SA Khalifa I Cacciotti I . A comprehensive review of the composition, nutritional value, and functional properties of camel milk fat. Foods. (2021) 10:2158. doi: 10.3390/foods10092158, 34574268 PMC8472115

[53] Dharmisthaben P Sakure A Liu Z Maurya R Das S Basaiawmoit B . Exploring mechanisms of novel antioxidative peptides from fermented camel milk (Kachchi breed, India) with anti-inflammatory activity in RAW 264.7 macrophage cell line. Int J Dairy Technol. (2023) 76:111–25. doi: 10.1111/1471-0307.12911

[54] Ayyash M Al-Dhaheri AS Al Mahadin S Kizhakkayil J Abushelaibi A. In vitro investigation of anticancer, antihypertensive, antidiabetic, and antioxidant activities of camel milk fermented with camel milk probiotic: a comparative study with fermented bovine milk. J Dairy Sci. (2018) 101:900–11. doi: 10.3168/jds.2017-13400, 29224862

[55] Abou-Soliman NHI El-Sayed MI Awad S. Antioxidant properties of fermented camel milk prepared using different microbial cultures. Food Nutr Sci. (2022) 13:861–77. doi: 10.4236/fns.2022.1311062

[56] Patel D Pinto S Pal M. A comprehensive review on the properties of camel milk and milk products. Int J Food Sci Agric. (2022) 6:200–7. doi: 10.26855/ijfsa.2022.06.010

[57] Abdelazez A Abd-Elmotaal H Abady G. Exploring the potential of camel milk as a functional food: physicochemical characteristics, bioactive components, innovative therapeutic applications, and development opportunities analysis. Food Mater Res. (2024) 4:e031. doi: 10.48130/fmr-0024-0020

[58] Saibhavana S Vasukhi SM Ramesh S Rajakumari R Abhijith AS Adithya Krishna S . Prospective nutritional, therapeutic, and dietary benefits of camel milk making it a viable option for human consumption: current state of scientific knowledge. J Exp Biol Agric Sci. (2023) 11:236–50. doi: 10.18006/2023.11(2).236.250

[59] Fernández-Tomé S Amigo L Hernández-Ledesma B Martínez-Villaluenga C. "Current evidence on the modulatory effects of food proteins and peptides in inflammation and gut microbiota". In: Martínez-Villaluenga C Peñas E, editors. Current Advances for Development of Functional Foods Modulating Inflammation and Oxidative Stress. London: Academic Press (2022)

[60] Guha S Sharma H Deshwal GK Rao PS. A comprehensive review on bioactive peptides derived from milk and milk products of minor dairy species. Food Prod Process Nutr. (2021) 3:2. doi: 10.1186/s43014-020-00045-7

